# A histone tale that enCOMPASSes pausing: new insights into the functional repertoire of H3K4me3

**DOI:** 10.1038/s41392-023-01529-x

**Published:** 2023-07-14

**Authors:** Thomas K. Albert, Kornelius Kerl

**Affiliations:** Department of Pediatric Hematology and Oncology, University Children’s Hospital Muenster, Muenster, Germany

**Keywords:** Cancer microenvironment, Tumour heterogeneity

A recently published study in *Nature* provides compelling evidence for the involvement of the histone H3K4me3 modification in regulating RNA polymerase II (Pol II) pause-release and elongation.^1^ The expansion of the functional repertoire of this active chromatin mark and the elucidation of the molecular mechanisms behind it represents a major advance for the field, not least because the epigenetic factors involved are linked to a wide range of human pathologies.

The term epigenetics derives from the Greek prefix “epi” and describes an inheritable regulatory state that manifests itself beyond the mere genetic information encoded in the DNA. Epigenetic regulation is imposed, among other things, at the level of DNA-packaging histone proteins, in particular via the reversible, covalent modification of their N-terminal tails. Although these post-translational modifications and their regulatory impact on gene expression were discovered and discussed as early as the 1960s, it was almost another 40 years before one of the leading figures in the field of epigenetics, the recently deceased David Allis, proposed his seminal model for the existence of a “code” of histone modifications. Each histone tail within a nucleosome can be decorated by a variety of (sometimes interdependent) modifications in different combinations. If only the number of publications were used as a benchmark, then lysine 4 tri-methylation in the tail of histone H3 is among the most important ones: as of May 2023, PubMed returns more than 3500 hits for the search term “H3K4me3”. The enzymes directly involved in H3K4me3 are the SET1/COMPASS histone methyltransferase complexes as “writers”, and the histone demethylases KDM5A/B as “erasers”. Since H3K4 methylation is generally associated with transcriptionally active genes, it is not surprising that mutations in SET1/COMPASS or KDM5 have been associated with tumorigenesis.^2,3^ However, the exact function of H3K4me3 is still a matter of debate, particularly with regard to the underlying molecular mechanisms responsible for transcriptional gene activation by Pol II. For example, there is also evidence that although transcription and H3K4me3 correlate well, the latter is ultimately not causal nor instructive for gene expression on a genome-wide scale.^4^

In this context, we refer to the aforementioned research article by Kristian Helin’s group, in which the authors present a comprehensive analysis of the effects of H3K4me3 on the early transcription elongation phase using cutting-edge genetic engineering in combination with an impressive arsenal of genome-wide analyses.^1^ To acutely deplete H3K4me3, the authors generated an inducible degron system that allows rapid ablation of either of two core subunits of mammalian SET1/COMPASS complexes, DPY30 or RBBP5, in mouse embryonic stem cells (mESCs). Chromatin immunoprecipitation-coupled sequencing (ChIP-seq) analysis of these cell lines revealed global loss of promoter occupancy by the two COMPASS factors as well as of H3K4me3 within 2 h after degron induction. The rapid turnover of H3K4me3 was dependent on KDM5 demethylase activity: a double knockout of murine Kdm5a and Kdm5b in the DPY30 degron cell line significantly delayed H3K4me3 depletion across all interrogated transcription start site (TSS) regions. Depletion of both COMPASS subunits also resulted in short-term reduction of mRNA synthesis, as demonstrated by time-course experiments using SLAM-seq, a metabolic labeling-sequencing technology for nascent mRNA with single nucleotide resolution. Again, the additional loss of KDM5 activity in DPY30 degron cells led to a significantly delayed decrease in mRNA synthesis. However, the negative effect of H3K4me3 depletion on de novo transcription initiation was neither the result of reduced preinitiation complex (PIC) formation nor reduced recruitment of the Pol II machinery to TSS regions. This finding per se is an important contribution to the long-standing debate as to whether H3K4me3 merely reflects transcriptional activity or whether it has a causal role in its regulation. However, the most important result of this work was yet to come: the rigorous proof that H3K4me3 affects a decisive post-initiation step, namely the arrest of Pol II.

After recruitment to gene promoters and subsequent PIC formation, Pol II initiates synthesis of a short nascent RNA, but then becomes stalled promoter-proximally before entering the gene body. It is now recognized that pausing is an universal hallmark of Pol II transcription, occurring at all protein-coding genes and other transcribed regions such as enhancers, and that the release of stalled Pol II into productive RNA synthesis is a pivotal checkpoint in gene expression.^5^ Through sophisticated multi-omics experiments using state-of-the-art technologies, the Helin group was able to demonstrate that H3K4me3 regulates both the pause-release and the elongation of Pol II.^1^ For example, by combining Pol II ChIP-seq experiments in degron cells with kinetic measurements of ongoing RNA synthesis of chromatin-engaged Pol II, they showed that H3K4me3 depletion led to a significant increase in the mean residence time of Pol II at promoter-proximal pause positions, while the elongation rate, i.e., the speed at which Pol II travels through the gene body, decreased.

To gain more mechanistic insights into this process, the authors used CRISPR/Cas9-based genome editing in COMPASS degron mESCs to proximity-tag the largest Pol II subunit RPB1, which enabled mass spectrometry-based identification of Pol II-associated proteins whose abundance would change upon loss of H3K4me3. One of these proteins is INTS11, the endonuclease subunit of the Integrator complex, which has been aptly described as key modulator of Pol II pausing and elongation before.^6^ To elucidate the exact role of INTS11, the authors created an additional degron variant of INTS11 in the background of the proximity-tagged RPB1/degron-tagged DPY30 mESC line. Acute loss of INTS11 in these cells led to increased Pol II pausing, broadly decreased productive elongation, and a global decrease in the expression of protein-coding genes and nascent mRNA synthesis. These data suggest H3K4me3-dependent recruitment of INTS11, which is required to enable transcriptional pause-release.

**Fig. 1:**
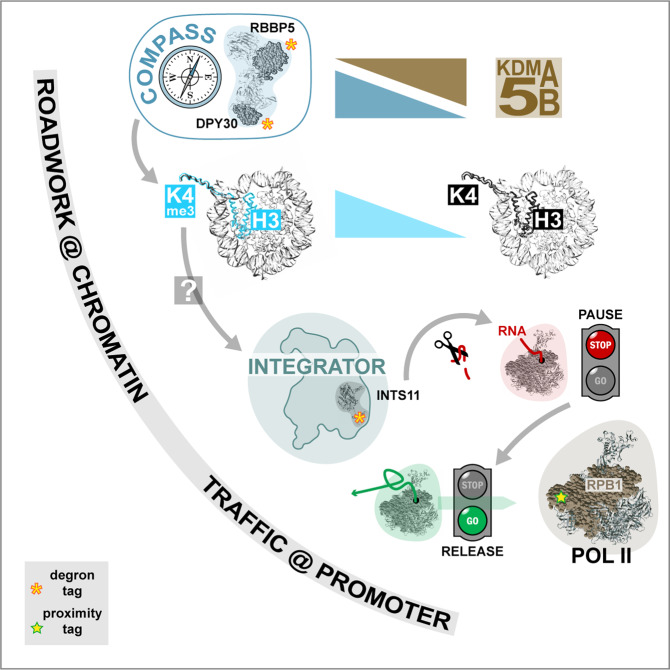
A model for the gene-regulatory role of H3K4me3. Shown are the key players at the chromatin level, the COMPASS complex as writer and KDM5A/B histone demethylases as eraser of histone H3 lysine 4 tri-methylation; and the key player at the transcription level, Pol II, which either pauses (red traffic light) or transitions into processive elongation (green traffic light) depending on the levels of H3K4me3 promoter decoration. The endonuclease INTS11 of the Integrator complex plays an essential role in this transition. The Integrator complex is recruited to H3K4me3-decorated nucleosomes in the promoters of active protein-coding genes by an as yet unknown mechanism. For further details see main text. The web-based iCn3D 3D structure viewer (https://www.ncbi.nlm.nih.gov/Structure/icn3d/) was used to create the structure images of COMPASS (PDB 6CHG), the nucleosome core particle (PDB 1AOI), INTS11 (PDB 7BFP), and Pol II (PDB 5FLM)

Taken together, these results lead to a model of H3K4me3-dependent coupling of chromatin “roadwork” with transcriptional “traffic” at the promoter level (Fig. 1). These epigenetic “street events” around the central axis of H3K4me3 can be summarized as follows: (1) H3K4me3 levels in promoter regions of active genes are accomplished by a dynamic equilibrium of COMPASS writer and KDM5 eraser functions; (2) H3K4me3 itself facilitates recruitment of the Pol II-associated Integrator complex (by an as yet unknown mechanism); and (3) Integrator is essential for the release of promoter-proximally paused Pol II via its INTS11 nuclease subunit.
